# Extramedullary Plasmacytoma of the Pancreas: A Rare Entity

**DOI:** 10.1155/2012/798264

**Published:** 2012-06-07

**Authors:** Alexis Smith, Hassan Hal, Elizabeth Frauenhoffer

**Affiliations:** ^1^Department of Radiology, Penn State Milton S. Hershey Medical Center, 100 University Drive, Hershey, PA 17033, USA; ^2^Department of Pathology, Penn State Milton S. Hershey Medical Center, 100 University Drive, Hershey PA 17033, USA

## Abstract

Extramedullary plasmacytomas are plasma cell neoplasms in organs other than the bone marrow. Most are found in the upper respiratory tract. Involvement of the pancreas is rare. We report a case of pancreatic plasmacytoma in association with advanced multiple myeloma.

## 1. Case

A 66-year-old African American woman first presented with left hip pain. MRI showed a left iliac wing tumor which was determined to be a plasmacytoma on subsequent CT-guided biopsy. She developed progressive disease over the next four years including lesions in multiple ribs and vertebral bodies, pelvis, skull, humeri, and femurs. She was treated with dexamethasone, thalidomide, and numerous courses of radiation, all with variable response.

**Figure 1:**
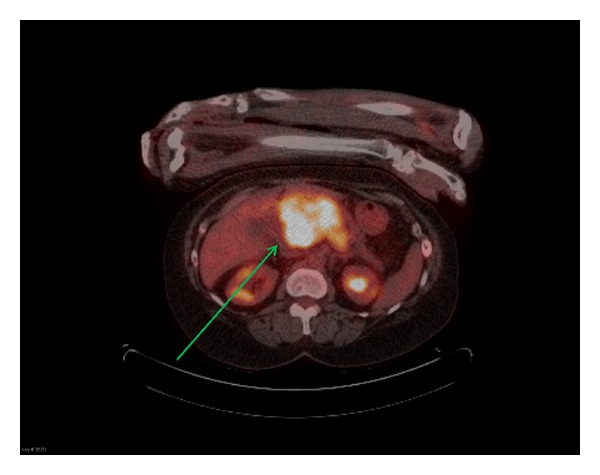
Axial PET/CT fusion image demonstrates a large, intensely FDG avid mass in the region of the pancreas (green arrow).

**Figure 2:**
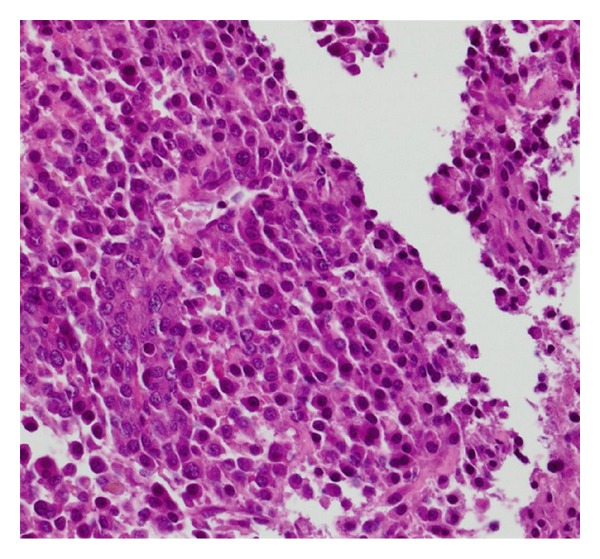
Core biopsy of the abdominal mass demonstrating sheets of atypical plasma cells which were positive with monoclonal kappa light chain staining by immunoperoxidases stains. No staining was seen for lambda light chains. (H&E stain, 20x magnification).

**Figure 3:**
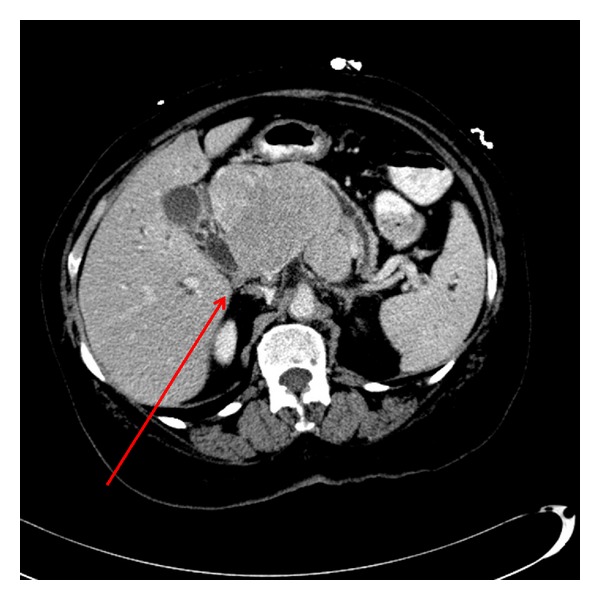
Contrast-enhanced axial CT image demonstrates a large homogeneously enhancing mass in the region of the pancreatic head which is causing compression of the distal common bile duct (red arrow).

The Patient was subsequently found to have elevated transaminases. On PET/CT, in addition to multiple bony lesions, there was an intensely FDG avid upper abdominal mass. The mass was in the region of the head and body of the pancreas with no definite pancreatic duct dilation (Figure 1). Biopsy confirmed multiple myeloma (Figure 2). The patient was admitted to the hospital two months later for jaundice with an elevated direct bilirubin due to the pancreatic mass compressing the distal common bile duct (Figure 3). She underwent a therapeutic ERCP with distal common bile duct stent placement which caused her jaundice to resolve. She then completed a second course of radiation and chemotherapy. Follow-up CT of the abdomen and pelvis showed significant reduction in the size of the pancreatic mass.

**Figure 4:**
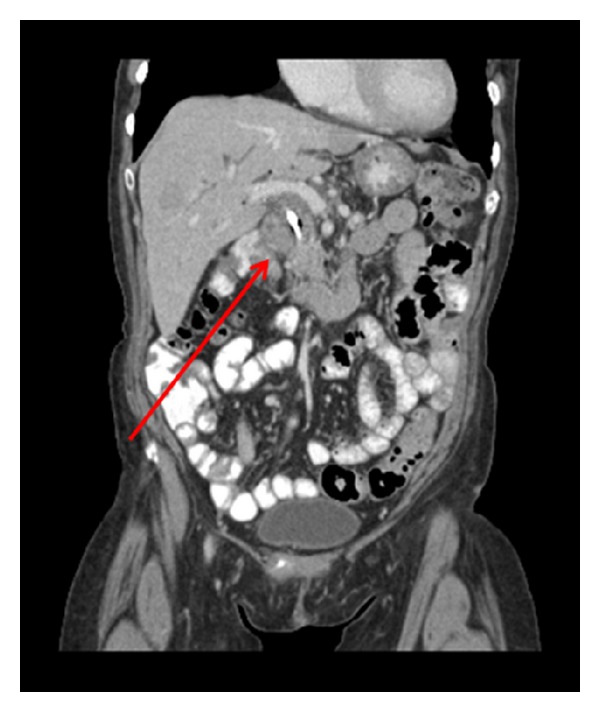
Contrast-enhanced coronal CT image demonstrates an ill-defined mass in the porta hepatis causing compression of the portal vein and common bile duct (red arrow).

Findings remained stable on subsequent studies until two years later when the patient developed nausea and abdominal pain. CT demonstrated interval enlargement of the pancreatic head mass. The patient received additional palliative radiation therapy; however follow-up studies showed multiple new metastatic liver lesions as well as an infiltrative mass in the porta hepatis encasing the portal vein, common hepatic artery and gastroduodenal artery (Figure 4). The porta hepatis mass appeared to extend from the pancreatic head mass. Since conventional therapy was no longer working, the patient was admitted into a clinical trial and expired four months later.

## 2. Discussion

Five percent of all plasma cell neoplasms involve organs outside the bone marrow [1]. These tumors are called extramedullary plasmacytomas. They are usually diagnosed after multiple myeloma of the bone marrow. Although the majority of extramedullary plasmacytomas involve the upper respiratory tract, ten percent occur in the gastrointestinal tract, primarily the liver, spleen, or stomach [2, 3].

There are approximately 25 case reports of pancreatic involvement in the English language literature. While they can develop in any part of the pancreas, many of these lesions are located in the pancreatic head. Diffuse enlargement of the pancreas has also been reported [3, 4]. Patients will typically present with abdominal pain and obstructive jaundice from compression of the common bile duct [5]. On CT, pancreatic plasmacytoma is commonly characterized as a homogeneously enhancing solid mass [6]. While this appearance is unlike that of a pancreatic adenocarcinoma, it can be difficult to distinguish from hypervascular neuroendocrine tumors. Percutaneous or open biopsy as well as endoscopic ultrasound-guided fine needle aspiration can be helpful in ruling out the other differential possibilities for a pancreatic mass including serous and mucinous tumors [7, 8].

Plasma cell tumors tend to be very radiosensitive and chemosensitive, so the treatment of choice for extramedullary plasmacytomas is generally some combination of surgery, chemotherapy and radiation therapy [9, 10]. ERCP with stenting of the biliary system is often performed for symptomatic relief of obstructive jaundice. Thalidomide and stem cell transplants are also currently under investigation [4]. One should always consider extramedullary plasmacytoma when a multiple myeloma patient presents with a mass.
